# 2-Methoxyestradiol, an Endogenous 17β-Estradiol Metabolite, Induces Antimitogenic and Apoptotic Actions in Oligodendroglial Precursor Cells and Triggers Endoreduplication via the p53 Pathway

**DOI:** 10.3390/cells13131086

**Published:** 2024-06-22

**Authors:** Sara. A. Schaufelberger, Martina Schaettin, Giovanna Azzarito, Marinella Rosselli, Brigitte Leeners, Raghvendra K. Dubey

**Affiliations:** 1 Department of Obstetrics and Gynaecology, Clinic for Reproductive Endocrinology, University Hospital Zurich, 8091 Zurich, Switzerlandgiovanna.azzarito@usz.ch (G.A.); marinella.rosselli@usz.ch (M.R.); brigitte.leeners@usz.ch (B.L.); 2 Zurich Centre for Integrative Human Physiology (ZIHP), University of Zurich, 8006 Zurich, Switzerland

**Keywords:** 2-methoxyestradiol, p53, glioblastoma tumor, endoreduplication, gliomas, apoptosis

## Abstract

The abnormal growth of oligodendrocyte precursor cells (OPCs) significantly contributes to the progression of glioblastoma tumors. Hence, molecules that block OPC growth may be of therapeutic importance in treating gliomas. 2-Methoxyestradiol (2ME), an endogenous tubulin-interacting metabolite of estradiol, is effective against multiple proliferative disorders. Based on its anti-carcinogenic and anti-angiogenic actions, it is undergoing phase II clinical trials. We hypothesize that 2ME may prevent glioma growth by targeting OPC growth. Here, we tested this hypothesis by assessing the impact of 2ME on the growth of an OPC line, “Oli-neu”, and dissected the underlying mechanism(s). Treatment with 2ME inhibited OPC growth in a concentration-dependent manner, accompanied by significant upregulation in the expression of p21 and p27, which are negative cell-cycle regulators. Moreover, treatment with 2ME altered OPC morphology from multi-arm processes to rounded cells. At concentrations of 1uM and greater, 2ME induced apoptosis, with increased expressions of caspase 3, PARP, and caspase-7 fragments, externalized phosphatidylserine staining/APOPercentage, and increased mitochondrial activity. Flow cytometry and microscopic analysis demonstrated that 2ME triggers endoreduplication in a concentration-dependent fashion. Importantly, 2ME induced cyclin E, JNK1/2, and p53 expression, as well as OPC fusion, which are key mechanisms driving endoreduplication and whole-genome duplication. Importantly, the inhibition of p53 with pifithrin-α rescued 2ME-induced endoreduplication. The pro-apoptotic and endoreduplication actions of 2ME were accompanied by the upregulation of survivin, cyclin A, Cyclin B, Cyclin D2, and ppRB. Similar growth inhibitory, apoptotic, and endoreduplication effects of 2ME were observed in CG4 cells. Taken together, our findings provide evidence that 2ME not only inhibits OPC growth and triggers apoptosis, but also activates OPCs into survival (fight or flight) mode, leading to endoreduplication. This inherent survival characteristic of OPCs may, in part, be responsible for drug resistance in gliomas, as observed for many tubulin-interacting drugs. Importantly, the fate of OPCs after 2ME treatment may depend on the cell-cycle status of individual cells. Combining tubulin-interfering molecules with drugs such as pifithrin-α that inhibit endoreduplication may help inhibit OPC/glioma growth and limit drug resistance.

## 1. Introduction

Oligodendrocytes represent a specialized brain cell population that play a key neuroprotective role by myelinating neurons/axons and facilitating optimal electrical conductance [1,2]. However, the abnormal growth of oligodendrocytes is also responsible for aggressive oligodendroglial neuronal/brain tumors, which constitute 5% of all neuroepithelial tumors at an incidence rate of a thousand new cases per year in the USA [3,4,5,6]. Gliomas, brain tumors of oligodendroglial or astroglial origin, are extremely difficult to treat due to their low response to DNA-alkylating agents, as well as the activation of pro-survival pathways. Myelinating oligodendrocytes evolve from oligodendrocyte precursor cells (OPCs), and are morphologically distinct with multiple tentacle- or arm-like processes [7,8,9,10]. The actin-rich growth cone of oligodendrocyte processes wraps around neurons/axonal segments to regulate cell–cell contact and myelination, and intracellular tubulin (microtubules) is critical for the extension of oligodendrocyte processes [1,2]. Functionally, microtubules and actin regulate the oligodendrocyte function of cell migration, as well as active cell debris clearing processes [1,2]. Targeting microtubules with microtubule-interfering agents has long been a therapeutic target against oligodendroglial cancer [11,12]. However, the development of resistance to these agents in cancer patients represents a major hurdle for the improvement of overall response and survival [13,14]. Moreover, the underlying mechanisms for drug resistance remain unclear. Since OPCs are involved in both neuroprotective and cancer/tumor pathophysiologies [1,2,10], a better understanding of the mechanisms driving their activity is required. Moreover, the role of endogenous molecules that modulate OPC activity may be of clinical importance.

2-Methoxyestradiol (2ME), an anticancer drug in phase II clinical trials, has been documented to have antiproliferative and antiangiogenic effects both in vitro and in vivo [15,16]. Although it is generally accepted that 2ME interacts with tubulin, resulting in faulty spindle formation and cell-cycle arrest, there is no evidence for a universal mechanism of action, and the mediators of apoptotic signaling by 2ME might even be cell-type-dependent. In this regard, 2ME protects against multiple proliferative disorders such as atherosclerosis, injury-induced neointimal thickening, glomerulosclerosis, pulmonary hypertension, endometriosis, and multiple cancers by targeting the mechanisms driving cell growth and death [15,16]. For example, 2ME induced apoptosis in ovarian carcinoma cells via the p38 and phospho-Bcl2 pathways [17]. In prostate cancer cells, cyclin B1 and cdc2 phosphorylation and their upstream regulatory molecular networks may be associated with 2ME-induced apoptosis and the inhibition of ERK-, JNK-, and p38-abrogated apoptosis in these cells [18]. In breast cancer cells, 2ME-induced apoptosis was associated with JNK activation and an increased phosphorylation of the antiapoptotic proteins Bcl-2 and Bcl-xL [19]. In sarcoma cells, the pro-apoptotic protein BAX, as well as the formation of ROS, were involved in the initial apoptotic event, and further 2ME treatment caused the upregulation of death receptor ligands FasL and TNF-α, and induced caspase-8 activation [20].

Taken together, the pleiotropic antitumor activities of 2ME, together with the fact that OPCs contribute to glioblastoma growth, suggest that 2ME might be a promising agent for the treatment of glioma, warranting an in-depth investigation into 2ME’s actions and underlying mechanisms in modulating OPC growth. The fact that 2ME was found to have moderate side effects in clinical phase I/II studies [21] and is effective in an orthotopic rat glioma model highlights its potential in treating gliomas [22]. It is therefore of therapeutic importance for future drug development to understand the mechanisms behind 2ME actions in relation to the oligodendrocyte cells of the brain.

Hence, the aim of our study was to assess the effects of 2-methoxyestradiol on an oligodendroglial cell line (Oli-neu cells), and to dissect the underlying mechanisms [23]. We chose this cell line as the model system since OPCs are implicated in glioblastoma growth and retain most OPC phenotypical characteristics even after several passages. Apart from their putative and widely studied role in glial repair, O-2A OLI-neu progenitor cells could be potentially involved in malignant glioma formation following genetic changes [24,25]. The OLI-neu cell line is an immortalized OPC line that was produced by transforming normal OPCs by overexpressing the oncogene known as *HER2* (also known as *EGFR-neu*). Thus, this cell line is a malignant cell line by definition since its growth is driven by the overexpression of a potent oncogene (for details, see Cellosaurus cell line Oli-neu, CVCL_IZ82). It is thus appropriate for our study, since 76% of glioblastomas are positive for *EGFR-neu*, whereas normal tissue expresses little if any *EGFR-neu* [26], together with the fact that Lindberg and colleagues used Oli-neu cells to demonstrate that OPCs can act as cells of origin for experimental glioma [24]. Additionally, we also conducted experiments on a O-2A progenitor cell line (CG4), which has been used to induce high-grade glioma [27]. Since 2ME is an investigational cytostatic drug intended for future use, the overall goal of our study was to investigate its impact on oligodendrocyte/OPC health, particularly at pharmacological concentrations of 2ME. To attain our goals, we assessed the following: whether 2ME inhibits OPC growth; whether 2ME influences OPC morphology; whether 2ME induces apoptosis in OPCs; whether 2ME triggers endoreduplication in OPCs; and the molecular mechanisms driving the growth inhibitory, apoptotic, and endoreduplication actions of 2ME. Our results show that, in OPCs, 2ME induced abnormalities in the cell cycle (endoreduplication), and mitosis eventually lead to cell death. Additionally, we show that 2ME-induced endoreduplication in OPCs involves p53.

## 2. Materials and Methods

### 2.1. Cell Culture

Oli-neu cells (OPCs): The culture dishes and flasks used to culture OPCs were coated with poly-D-lysine solution (5 mg/mL in H_2_O). After coating the surface for 30 min with poly-D-lysine solution, the solution was removed, and the coated surface rinsed with sterile water prior to the addition of culture medium to the flasks or plates. Freshly defrosted OPCs were plated in 6 cm culture dishes and grown to confluence. Subsequently, the cells were dislodged by trypsinization and transferred to a T 75 flask. OPCs were plated at a density of 1 or 1.3 million cells in 10 cm plates or in T75 flasks, respectively. Undifferentiated OPCs were expanded in SATO medium (DMEM containing 1% FCS, 25 µg/mL gentamicin, 10 µg/mL transferrin, 0.5 µM L-thyroxine, 222 nM Na-selenite, 10 g/mL insulin, 16.1 µL/mL putrescine, 62 ng/mL progesterone, 2 mM L-glutamine, 1 mM pyruvate, and 340 ng/mL tri-iodo-thyrodine) and PGDF (20 ng/mL), or in DMEM/F12 medium supplemented with B27 (DMEM/F12 containing 1% penicillin/streptomycin, 2% B27 supplement [with vitamin A], and 1% glutamine and 20 ng/mL PDGF). To test whether the undifferentiated OPCs differentiated to oligodendrocytes, OPCs were treated with 1 mM dibutyryl cAMP for 3 days. To confirm the progenitor nature of the OPCs, they were characterized by immunostaining for the presence and/or absence of GD3 (b series disialoganglioside), O4 (oligodendrocyte marker), and GFAP (glial fibrillary acidic protein).

CG4 cells were cultured and expanded under culture conditions reported to maintain their progenitor phenotype [28]. In this context, CG4 cells were maintained in poly-D-lysine-coated flasks (BD) using a complete growth medium (GM) of DMEM with high glucose, glutamine, and sodium pyruvate, and 50 U/mL penicillin/streptomycin (Life Technologies Corp. Grand Island, NY, USA), plus the following additives (final concentration), from Sigma-Aldrich (St. Louis, MO, USA) unless otherwise indicated: insulin (10 µg/mL; CellGro, Manassas, VA, USA); transferrin (25 µg/mL); sodium selenite (5.2 ng/mL); putrescine (1 µg/mL); progesterone (6.3 ng/mL); biotin (2.4 ng/mL); triiodothyronine (sodium salt) (20.2 ng/mL; Calbiochem, Gibbstown, NJ, USA); hydrocortisone (7.25 ng/mL); BSA Fraction V (0.1%; Life Technologies Corp. Grand Island, NY, USA); 1x trace elements (Corning, Mediatech Inc. Manassas, VA, USA); and PDGF-AA and b-FGF (10 ng/mL each; Peprotech, Rocky Hill, NJ, USA). Both growth factors were replaced every 2 days at a concentration of 20 ng/mL.

### 2.2. Protein Concentration Assay

Protein concentrations were determined by colorimetric assay using a bicinchoninic acid (BCA) protein assay kit (Pierce, Thermo Fisher Waltham, MA, USA (23227)) according to the manufacturer’s protocol. The absorbance was measured with a SpectraFluor Plus (Fluorescence Reader, Tecan Salzburg, Austria) and analyzed with the Magellan 6 software.

### 2.3. Western Blotting

Western blot analysis was performed with whole-cell lysates. Cells were grown on 6 cm dishes, washed twice with HBSS with Ca^2+^ and Mg^2+^, and lysed by the addition of 100 µL lysis buffer (Cell Signaling Technology, Danvers, USA) containing 20 mM Tris (pH 7.5), 150 mM NaCl, 1 mM ethylenediaminetetraacetic acid (EDTA), 1 mM ethylene glycol tetraacetic acid (EGTA), 1% triton X-100, 2.5 mM sodium pyrophosphate, 1 mM -glycerophosphate, 1 mM sodium vanadate (Na_3_VO_4_), 1 g/mL leupeptin, 0.287 mM phenylmethanesulfonyl fluoride (PMFS), and 0.2% sodium dodecyl sulphate (SDS). The cell lysate was scraped from the dishes and homogenized by sonication (thrice, for 3 s each). The protein concentration was determined spectrophotometrically using a bicinchoninic acid protein assay kit from Pierce (Thermo Fisher Waltham, MA, USA (23227)) and SpectraFluor Plus (Tecan, Salzburg, Austria).

Equal amounts of proteins (20–25 µg per lane) were diluted with 5X loading buffer with 0.313 mM Tris/HCl, pH 6.8, 10% SDS, 0.05% bromophenol blue, 50% glycerol (Fermatas, Hanover, MD, USA (R0891)), and 0.1 M dithiothreitol (DTT). Thereafter, samples were denatured at 95 °C for 5 min. After separation in 10% SDS-polyacrylamide gel, the proteins were transferred to a nitrocellulose membrane. Successful transfers were visualized by Ponceau S staining solution (2%, Sigma-Aldrich, St. Louis, MO, USA (P-7767)). After two seconds, the Ponceau S solution was transferred back to the vial and the membrane was washed in PBS (phosphate-buffered saline) until the red staining disappeared. When necessary, the membranes were cut at the appropriate molecular weight before the Ponceau staining was washed out. Membranes were blocked in 5% nonfat dry milk in PBS/0.2% Tween 20 either overnight at 4 °C or at RT for 1 h. After blocking, the membrane was incubated with the primary antibodies (1–4 h at RT or overnight at 4 °C). Primary and secondary antibodies were dissolved in 1% nonfat dry milk in PBS containing 0.2% Tween 20. To remove unbound primary antibodies, the membrane was washed three times for 10 min each, with 1% milk in PBS/0.2% Tween 20. Incubation with peroxidase-conjugated secondary antibodies was again performed either for 1–4 h at RT or overnight at 4 °C. The membrane was washed one time with 1% nonfat dry milk in PBS/0.2% Tween 20 and two times with PBS/0.2% Tween 20. Peroxidase activity was detected using ECL (Pierce) and the membranes were exposed to X-OMAT LS films. When the membranes were analyzed with the LI-COR system, all steps were performed as described above, except that the membranes were blocked in 5% nonfat dry milk in PBS without Tween and incubated with the secondary antibodies (IRDye 680/800 conjugated goat-rabbit IgG) for 45 min, and thereafter washed with PBS without Tween 20. For the successive detection of different proteins on the same membrane, the membrane was washed with PBS/0.2% Tween 20 after the analysis of the first protein, incubated for 20 min with stripping buffer 1 (0.1 M glycine in PBS, pH 2–3), and subsequently shortly washed in stripping buffer 2 (1 M NaCl in PBS). Subsequently, the membranes were washed three times with PBS/0.2% Tween 20.

### 2.4. Growth Studies

Studies were conducted using phenol red-free medium. When not specified, cells were growth-arrested for 24 h in medium containing 0.1% steroid-free FCS. The pretreatment of cells was performed by adding the compound of interest during starvation. Cell growth was induced by the addition of 2.5% steroid-free FCS in the presence or absence of 0–10 µM of 2ME. In all cell-growth experiments, cell growth was analyzed by 3H-thymidine incorporation and by counting cell numbers. For the experiments incorporating 3H-thymidine, cells were treated with 2.5% steroid-free FCS plus treatments for 20 h, and subsequently fresh treatment medium was added with 1.0 µM Ci/well ^3^H-thymidine for 4 h. Aliquots from 4 different wells per treatment were mixed with 10 mL scintillation fluid and counted in a liquid scintillation counter. For cell number experiments, sub-confluent cells were growth-arrested for 24 h and subsequently treated for 24 h, as mentioned above. After 24 h, the cells were dislodged by trypsinization and counted in a coulter cell-counter.

### 2.5. FACS Analysis

For cell-cycle distribution analysis, 0.4 million cells were plated in growth medium into 10 cm dishes. After attachment overnight, the cells were washed once with PBS and the medium was replaced with medium containing different 2ME concentrations (0–10 µM). After incubation at 37 °C and 5% CO_2_ for two days, cells were washed twice with HBSS without Ca^2+^ and Mg^2+^ and harvested by trypsinization. After the addition of an equal amount of medium, cells were centrifuged for 10 min at 1250 rpm and 4 °C. The pellet was re-suspended in 1 mL sample buffer (1 g/L glucose in PBS). Cells were counted and sample buffer was added until the cell number reached a concentration of 1 million cells per mL. The cell suspension (1 mL) was centrifuged in a FACS tube for 10 min at 1350 rpm and 4 °C. After the removal of the supernatant, cells were fixed by the dropwise addition of ice-cold 70% ethanol and stored at 4 °C until analysis. For DNA staining with propidium iodide (PI), the fixed cells were centrifuged for 7 min at 2600 rpm and 4 °C. Subsequently, the pellet was resuspended in 0.5-1 mL PI-staining solution (50 µg/mL propidium iodide, 100 U/mL RNase A, sample buffer) and the tubes were shaken for 0.5–1 h at RT in the dark. The DNA amount was analyzed by flow cytometry.

### 2.6. Metabolic Activity Using MTT Assay

Cells were grown overnight in a 96-well plate (10,000 cells per well). Subsequently, the attached cells were treated with 2ME (5 µM) for 18 h under standard tissue culture conditions. After the treatment period, the medium was replaced by fresh media containing 0.5 mg/mL MTT and the test compound, and the cells were incubated for an additional 2 h. Subsequently, the medium was aspirated and the cells were lysed by the addition of 200 µL DMSO, and the absorbance was measured at 540 nm using a Spectrofluorometer Plus reader (Tecan Group Ltd., Salzburg, Austria). In cells treated identically in parallel, cell number was also quantified and used to calculate the metabolic activity per cell.

### 2.7. Apoptosis Studies

To assess the apoptotic impact of 2ME in OPCs, cells plated in culture dishes were grown to sub-confluence and treated with or without 0 to 10 µM of 2ME for 48 h under standard tissue culture conditions. Subsequently, the OPCs were lysed and the lysates analyzed by Western blotting for caspase-mediated mechanisms by assessing caspase-3 and caspase-7 fragmentation. Moreover, the involvement of PARP was assessed by measuring PARP fragmentation. Staining with APOPercentage dye, a specific marker for apoptosis which stains externalized phosphatidylserines was used to assess whether 2ME has apoptotic or necrotic actions on OPCs. Briefly, as per the manufacturer’s instructions for the APOPercentage™ kit (Biocolor, Belfast, Northern Ireland), OPCs grown in 24-well culture plates and treated with or without 2ME (0–10 µM) or H_2_O_2_ (25 mM; positive control) for 20 h were stained with the APOPercentage dye for 1 h. The medium was then carefully aspirated, and cells were washed twice with PBS. Three representative areas from each well were photographed.

### 2.8. Microscopy (Bright Field, Fluorescence, and Live)

For the analysis of DAPI-stained OPCs, the cells were grown on coverslips coated with poly-D-lysine. After the treatment with 2ME for the indicated times, cells were fixed by slowly adding fresh ethanol plus acetic acid mixture to the cell (7.5 mL EtOH 100 puriss, 2.5 mL acetic acid), and then waiting until it evaporation. Thereafter, 10 µL of DAPI counterstain was added on top of the cells, and the fluorescence was analyzed using the Olympus Microscope BX61.

For the live microscopy, cells treated with or without 2ME and observed and/or live images were recorded for subsequent analyses using an Olympus IX81 microscope. Time-lapse phase-contrast imaging of the effects of 2ME on OPN cell activation and fusion was recorded. Live videos were recorded following the 48 h treatment of cultured OPCs with 2ME (5 or 10 µM) using an Olympus IX81 microscope. To analyze the effects of 2ME on OPCs, cells were plated on pre-coated 6-well plates at a density of 200,000 cells/well and allowed to attach and grow for 24 h. Subsequently, the cells were treated with 2ME for 24 or 48 h in the presence of complete OPN growth medium, and the effects were observed by employing phase-contrast microscopy.

### 2.9. Materials

Detail for Material used can be downloaded at: https://www.mdpi.com/article/10.3390/cells13131086/s1, Table S1: Materials used.

### 2.10. Statistical Analysis

Data were analyzed using ANOVA, and statistical significance (*p* < 0.05) was calculated using Fisher’s least significant difference test.

## 3. Results

### 3.1. Cultured OPCs

**Figure 1:**
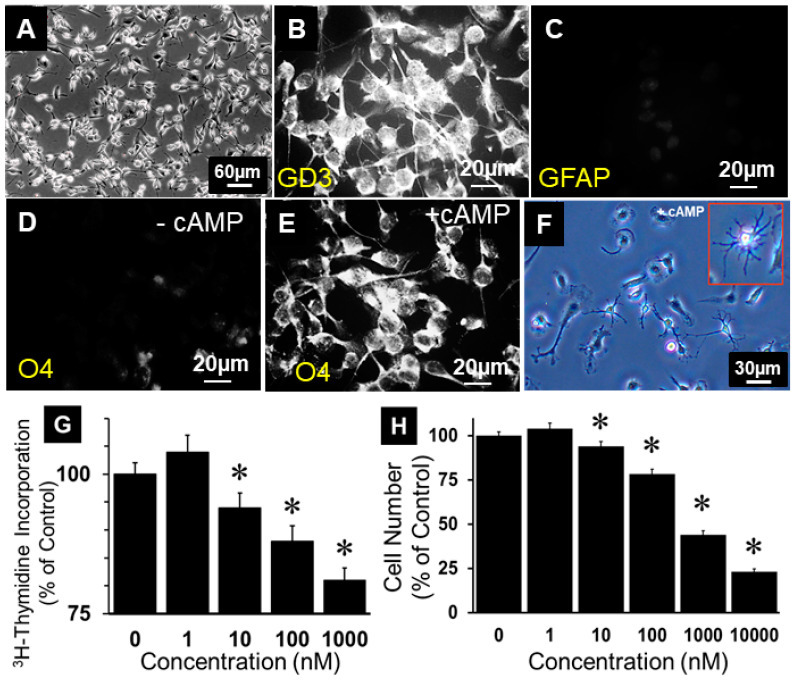
Growth-inhibitory effects of 2-methoxyestradiol (2ME) in Oli-neu (OPCs) cells. (**A**) Representative photomicrograph showing cultured OPCs (4× mag). (**B**) Immunostaining of OPCs depicting positive staining for GD3 and negative staining for GFAP (**C**). Immunostaining of undifferentiated OPCs showing little or negligible staining for O4 (**D**), but positive O4 staining in OPCs treated with 1 mM dibutyryl cAMP (1 mM) for 48 h to differentiate to oligodendrocytes (**E**). Panel (**F**) depicts representative photomicrograph of OPCs after treatment with 1 mM dibutyryl cAMP (1 mM) for 48 h with characteristic oligodendrocyte morphology at a higher magnification (100×). (**G**) Bar graph showing the concentration-dependent inhibitory effects of 2ME on FCS (2.5%)-induced DNA synthesis, measured by assessing 3H-thymidine incorporation. (**H**) Bar graph depicting the inhibitory actions of 0–10 µM 2ME on 2.5% FCS-induced changes in cell (OPC) number. Each data point represents mean ± SEM from 3 separate experiments, each conducted in triplicate or quadruplicate; * *p* < 0.05 vs. vehicle-treated control. Panel (**B**–**E**) magnification 100×.

Cultured oligodendroglial cell lines known as “Oli-neu”, which represented OPCs and were established from O2A progenitor cells [23], were used in the present study. We chose this cell line as the model system to investigate the growth effects of 2ME as it retains most of the phenotypical and electrophysiological characteristics of the cell even after several passages. As shown in Figure 1A, undifferentiated OPCs grew robustly in culture and retained their characteristic bipolar arm/tentacle-like processes. Undifferentiated OPCs stained positive for GD3 and were negative for O4, as well as GFAP (Figure 1B–D), confirming they were undifferentiated OPCs. These cells were further used for the experiments. Treatment with cAMP for 3 days triggered O4 expression (Figure 1E) and differentiation to oligodendrocytes with multiple arms/tentacles (Figure 1F).

### 3.2. 2ME Inhibits Oli-Neu Cell Growth

To investigate whether 2ME, a major endogenous E2 metabolite, is able to regulate OPC growth, we assessed the concentration-dependent effects of 2ME on 2.5% SF-FCS-induced DNA synthesis and cell number. Treatment with 1–10,000 nM 2ME significantly and concentration-dependently inhibited DNA synthesis in a concentration-dependent fashion. At concentrations of 10, 100, and 1000 nM, 2ME abrogated FCS-stimulated growth from 100 ± 0.8% to 91.1 ± 1.1% (*p* < 0.05), 86.8 ± 1.4% (*p* < 0.05), and 76.3 ± 1.7% (Figure 1G) (*p* < 0.05). Similarly, treatment with 10, 100, 1000, and 10,000 nM 2ME significantly decreased cell number in a concentration-dependent manner from 100 ±1.5% to 97.0 ± 0.8%, 90.0 ± 0.4%, 70.7 ± 0.5%, 44 ± 1.5%, and 23 ± 1.8% (Figure 1H) (*p* < 0.05).

### 3.3. 2ME Modulates p21 and p27 Expression

**Figure 2:**
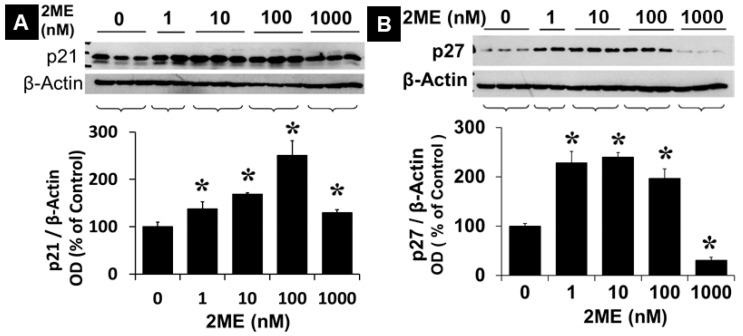
Modulatory effects of 2ME on p21 and p27 expression. Representative Western blot showing the effects of 2ME on the expression of p21 (**A**) and p27 (**B**) in Oli-neu cells. Cells were serum-starved for 24 h in SATO medium containing 0.25% steroid-free FCS, without phenol red and without progesterone. Subsequently, cells were treated with 1–1000 nM 2ME for 48 h in SATO containing 1% steroid-free FCS, without phenol red and without progesterone. The bar graphs show the densitometric analysis of the changes observed, normalized to the internal standard, β-actin. The results are presented as mean ± SEM (*n* = 3). * *p* < 0.05 vs. control.

To determine the mechanisms by which 2ME inhibits cell proliferation, we examined the expressions of p21 and p27, which negatively regulate cell-cycle progression and inhibit growth. Treatment with 10, 100, and 1000 nM 2ME significantly increased p21 expression from 100 ± 9.4% to 169 ± 2.1%, 250 ± 30.6%, and 129.8 ± 6.2% (Figure 2A) (*p* < 0.05). Similar to p21, treatment with 1, 10, and 100 nM 2ME significantly increased p27 expression from 100 ± 5.1% to 228.4 ± 22.7%, 239.8 ± 9.5%, and 196.8 ± 19.6% (Figure 2B) (*p* < 0.05). Interestingly, treatment with 1000 nM 2ME, which induced pro-apoptotic actions, significantly decreased p27 expression to 30.8 ± 5.9% (*p* < 0.05). Similar to p27, at concentrations higher than 1 µM, 2ME lowered the magnitude of p21 expression from 250% at 100 nM to 130% and control levels.

### 3.4. 2ME Modulates OPC Morphology

**Figure 3:**
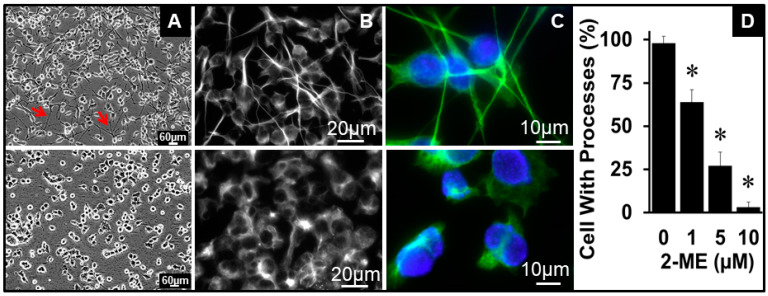
Morphological changes induced by 2ME in Oli-neu cells (OPC). Representative phase-contrast ((**A**); red arrows indicate cell arm/tentacles magnification 4×) and bright-field fluorescent (**B**,**C**) images showing retraction of OPC arms/tentacles in OPCs treated with 2ME (5 µM) for 24 h (magnification 40× and 100×). DAPI was used for nuclear staining (blue). (**D**) Bar graph depicts the concentration-dependent effects of 2ME on OPC arm retraction. Cells at 10 different locations of the photomicrograph were observed and counted to assess the effects of 2ME on cell rounding or loss of tentacles. * *p* < 0.05 vs. vehicle-treated control.

Cultured OPCs have a specific morphology with tentacle- or arm-like processes (Figure 1B). Since these processes play a key role in regulating OPC function, we assessed how 2ME impacts this characteristic. As shown in the representative phase-contrast and immunofluorescence photomicrographs in Figure 3A–C, the treatment of OPCs with 2ME influenced OPC morphology and contracted the arm/tentacle processes in a concentration-dependent fashion (Figure 3D). At a concentration of 5 µM of 2ME, most cells lost their elongated arm-like processes and became rounded (Figure 3C).

### 3.5. 2ME Treatment Induces OPC Metabolic Activity

**Figure 4:**
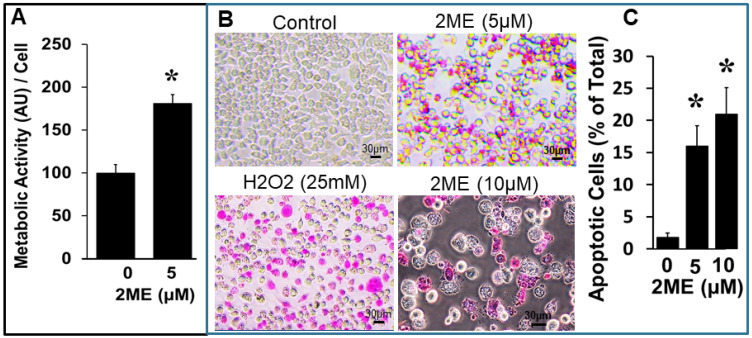
Treatment with 2ME induces metabolic activity and apoptosis in Oli-neu (OPC) cells. (**A**) Bar graph depicting the effects of 5 µM 2ME on OPC metabolic activity measured by MTT assay and normalized to OPC numbers counted after treatment with vehicle or 5 µM 2ME. The graph represents the metabolic activity per cell. Data represent mean ± SEM (*n* = 3 each in triplicates). * *p* < 0.05 vs. control. (**B**) Representative photomicrographs stained with the APOPercentage dye, a specific marker for apoptosis. OPCs treated with H_2_O_2_ (25 mM) served as positive control for apoptosis (magnification 40×). (**C**) Bar graph depicting percentage of apoptotic cells (APOPercentage) in response to 2ME (mean ± SEM *n* = 3). * *p* < 0.05 vs. control.

Since 2ME inhibited OPC growth, we next assessed its impact on OPC viability using a MTT assay, which detects mitochondrial metabolic activity as a measure for viable cells. In OPCs treated with 5 µM 2ME in normal growth medium for 18 h, metabolic activity was assessed by measuring the absorbance at 540 nm. In parallel, the cell number after treatment with 5 µM 2ME was assessed by cell counting. Although treatment with 2ME decreased cell number, it significantly increased the calculated metabolic activity per cell from 100 ± 10.0% to 181.1 ± 10.3% (Figure 4A) (*p* < 0.05). 

### 3.6. 2ME Treatment Induces Apoptosis

To assess whether 2ME induced cell death by apoptosis or necrosis, 2ME-treated cells were stained with the APOPercentage dye, which stains externalized phosphatidylserines. As shown in Figure 4B, the treatment with 2ME (5 and 10 µM) significantly increased the number of cells positively stained for externalized phosphatidylserines from 100 ± 1.0% to 470 ± 13.3% and 617.3 ± 14.5% (Figure 4C) (*p* < 0.05).

**Figure 5:**
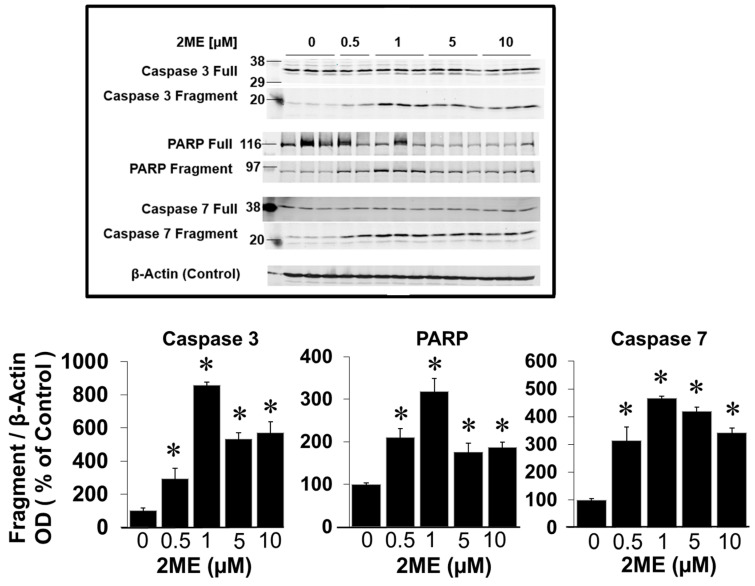
Western blots showing the modulatory effects of 2ME on caspase 3 and caspase 7 cleavage, as well as on PARP fragmentation in Oli-neu cells. The bar graphs depict the densitometric analysis of the changes observed in cleavage products or fragments, normalized to the total protein amount. The results are presented as mean ± SEM (*n* = 3). * *p* < 0.05 vs. control.

**Figure 6:**
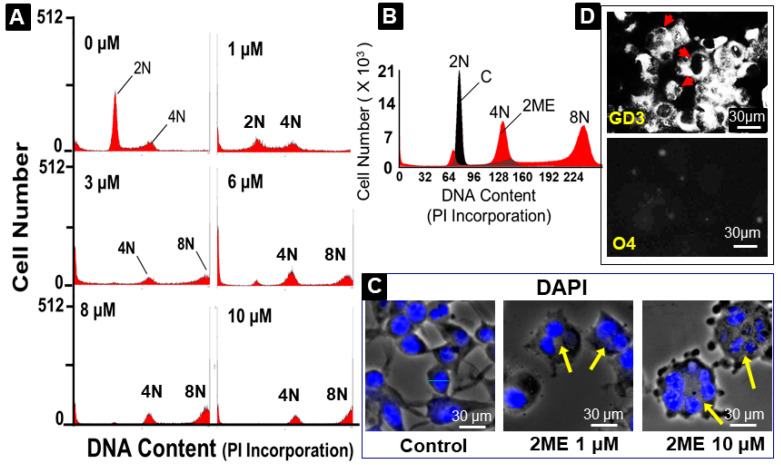
Endoreduplication-promoting effects of 2ME in Oli-neu (OPCS) cells. FACS analysis of PI-stained OPCs (**A**,**B**). Fluorescent microscopy of DAPI-stained cells (**C**) showing that treatment with 2ME induces endoreduplication in OPCs and positively stains for GD3, but not O4 (**D**). Panel (**A**) depicts OPCs treated with 1–10 µM 2ME for 48 h. The DNA was stained with propidium iodide prior to FACS analysis. Appearance of 8N cell population is evident in representative FACS of 2ME-treated OPCs. Panel (**B**) compares normal and 2ME-treated OPCs by overlapping untreated OPCs (black) and 2ME-treated OPCs (red). Panel (**C**) depicts DAPI-stained multinucleated OPCs (yellow arrows) following 2ME treatment (magnification 100×). Panel (**D**) shows multinucleated cells (red arrows) stained positive for GD3 and negative for O4 (magnification 40×).

To assess the role of caspases 3 and 7 in the 2ME-mediated apoptosis in OPCs, we analyzed their fragmentation by Western blotting. Treatment with 0–10 µM 2ME significantly induced the fragmentation of caspase 3 from 100 ± 12.2% to 192.2 ± 8.5%, 234.3 ± 26.9%, and 271.3 ± 26.1% (Figure 5) (*p* < 0.05). Similar to caspase 3, caspase 7 fragmentation was significantly induced by treatment with 1.25, 2.5, and 5 µM 2ME from 100 ± 27.3% to 316.5 ± 28.8%, 319.6 ± 35.4%, and 303.5 ± 35.2% (Figure 5) (*p* < 0.05). Furthermore, treatment with 1.25, 2.5, and 5 µM 2ME significantly increased PARP fragmentation from 100 ± 40.0% to 1167.9 ± 228%, 655.4 ± 106.8%, and 622.8 ± 159.5% (Figure 5) (*p* < 0.05). Consistent with the activation of pro-apoptotic mechanisms, in OPCs incubated with 5 µM 2ME for 48 h, DNA staining with PI (propidium iodide: a general marker for cell death) followed by the FACS analysis of the PI-stained cells showed that 2ME induces cell death in OPCs. As shown in Figure 6, treatment with 2ME led to a shift and decrease in the number of cells with 2N DNA content, probably due to nuclear fragmentation.

### 3.7. 2ME Treatment Induces Endoreduplication

As a next step, we assessed DNA quantity per cell by measuring PI incorporation, as well as DAPI staining. To this end, cells were grown to subconfluency in normal SATO medium. Thereafter, the cells were treated with 0–10 µM 2ME for 48 h and PI incorporation was assessed by FACS analysis. As shown in Figure 6, treatment with 2ME > 1 µM concentration resulted in a high percentage of cells containing a DNA content of >4N, indicating that cells skipped mitotic cell division, i.e., underwent endoreduplication. To confirm this, cells treated with and without 2ME for 48 h were examined by fluorescence microscopy after DAPI staining. As shown in Figure 6, 2ME-treated cells were rounder and bigger than non-treated cells and they contained multiple nuclei (see arrows). Moreover, the immunostaining of the multinucleated cells was positive for GD3 and negative for O4 (panel D). Negative staining was also observed for GFAP, suggesting that 2ME induces endoreduplication in OPCs.

### 3.8. Mechanism Underlying 2ME-Induced Endoreduplication

**Figure 7:**
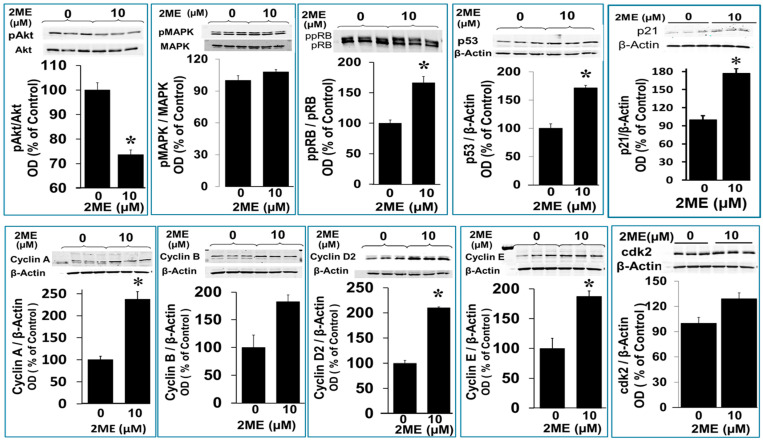
Western blots depicting the modulatory effects of 2ME on various cell-cycle and signal-transducing proteins associated with endoreduplication. OPCs were treated with 10 µM 2ME for 48 h and the changes in pAkt, pMAPK, ppRB, p53, p21, cyclin A, cyclin B, cyclin D2, cyclin E, and cdk2 were assessed in the lysates. Representative blots and bar graphs depict the effects of 2ME compared to vehicle-treated control. The modulatory actions are presented as mean ± SEM (*n* = 3). * *p* < 0.05 vs. control.

As shown above, 2ME treatment induced endoreduplication, and this was accompanied by an increased metabolic activity per cell. To identify the molecular changes induced by 2ME treatment, we assessed the phosphorylation status of Rb and MAPK, which are both known to be involved in proliferation. To this end, Oli-neu cells were grown to subconfluency and treated with or without 10 µM 2ME for 48 h. Protein phosphorylation was assessed by Western blot analysis. Rb acts as a tumor suppressor by providing a cell-cycle checkpoint between the G1 and S (synthesis) phases. The antibody used in this study binds to phosphorylated as well as hyperphosphorylated Rb. The active, underphosphorylated form (pRb) is primarily found in resting or fully differentiated cells, while the hyperphosphorylated form (ppRb) is primarily found in proliferating cells. pRb inactivation by phosphorylation is a critical step leading to S-phase commitment at the G1 checkpoint of the cell cycle. As shown in Figure 7, less pRb was observed in cells treated with 2ME compared to the control. Therefore, the ratio between pRb and ppRb increased due to the treatment with 2ME, indicating that treatment with 10 µM 2ME promotes proliferation. Another protein which is known to play a key role in proliferation is MAPK. The phosphorylation of MAPK, however, was not influenced by the treatment with 2ME. Importantly, 2ME downregulated pAkt expression from 100 ± 3.65% to 73 ± 1.7% (*p* < 0.05) (Figure 7).

Furthermore, cyclins are a family of proteins that control the progression through the cell cycle by activating Cdk enzymes. There are several cyclins that regulate cell-cycle progression and they are active in different phases. We assessed the impact of 2ME on G1/S cyclins, which control cell-cycle progression at the G1/S transition by activating Cdk enzymes. Cyclin A is active during the G1 phase, while cyclin D and E regulate the transition from the G1 to the S phase. G2/M cyclins are essential for the control of the cell cycle at the G2/M transition, where cyclin B regulates the progression from the S to the G2 phase. Treatment with 10 µM 2ME for 48 h resulted in an increase of the expression of cyclin A from 100 ± 7.7% to 237.8 ± 17.5%, of cyclin B from 100 ± 22.4% to 182.7 ± 12.3%, of cyclin D2 from 100 ± 5.5% to 209.0 ± 2.1%, and of cyclin E from 100 ± 17.2% to 187.4 ± 9.1%, as well as a minor change in cdk2 from 100 ± 4.1 to 127 ± 3.45% (Figure 7) (*p* > 0.05).

**Figure 8:**
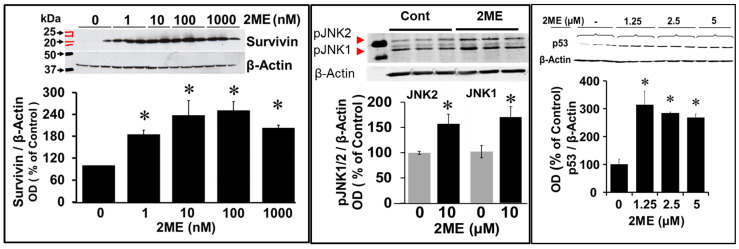
Western blots depicting the modulatory effects of 2ME on various signaling proteins associated with endoreduplication. OPCs were treated with 0–10 µM 2ME for 48 h and the changes in JNK1, JNK2, survivin, and p53 expression were assessed in the lysates. Representative blots and bar graphs depict the effects of 2ME compared to the vehicle-treated control. The bar graphs depict the modulatory actions of 2ME, presented as mean ± SEM (*n* = 3). * *p* < 0.05 vs. control.

Finally, p53 is known to growth-arrest cells at the G1/S regulation point in the case of DNA damage. When DNA repair proteins are able to overcome the DNA damage, the cell continues the cell cycle. In the case of irreparable DNA damage, p53 initiates apoptosis. The expression of p53 was analyzed by Western blot analysis after 48 h with or without treatment with 1.25 to 10 µM 2ME. As shown in Figure 7, p53 expression was significantly upregulated by 2ME from 100 ± 8.0% to 171.5 ± 4.5% (*p* < 0.05) at 10 µM; moreover, the upregulation was ≈300% at 1.25 to 5 µM of 2ME (Figure 8). 

Next, we assessed the impact of 2ME on survivin, JNK1, and JNK 2. As shown in Figure 8, the treatment of OPCs with 1–10 µM 2ME significantly induced survivin expression in a concentration-dependent fashion with a maximal increase of ≈250% at the concentration of 0.1 µM. Moreover, treatment with 2ME (10 µM) significantly induced phosphorylated JNK1 and JNK2. Similarly, treatment with 0–5 µM 2ME induced p53 expression in the OPCs by ≈300% (Figure 8).

**Figure 9:**
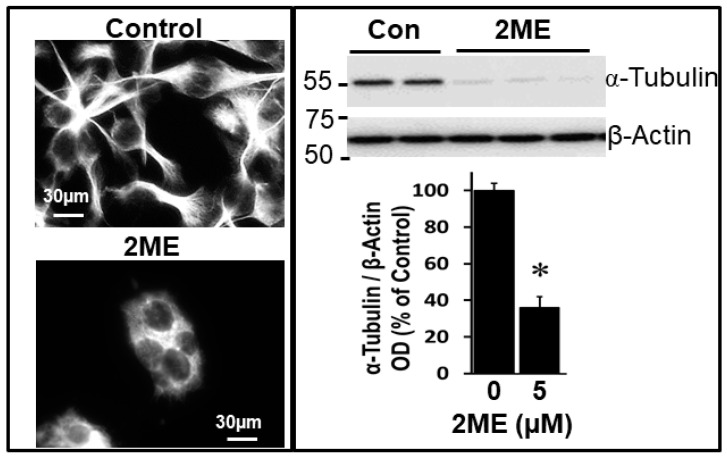
Photomicrographs and Western blots showing the inhibitory actions of 2ME on tubulin polymerization in OPCs. Cells were treated with 5 µM 2ME for 48 h and the changes in α-tubulin were assessed in the lysates by Western blotting, as well as by immunostaining fluorescence microscopy (magnification 40×). Representative photomicrographs depict the reduction in acetylated α-tubulin staining in OPCs treated with 2ME compared to the untreated control. Western blots also depict the effects of 2ME compared to the vehicle-treated control. Immunostaining was observed in 6 separate samples and Western blots in duplicate or triplicate. * *p* < 0.05 vs. control.

Interference with tubulin polymerization is known to play a key role in mediating the anti-proliferative actions of 2ME. By using antibodies to acetylate α-tubulin, we assessed whether 2ME interferes with tubulin polymerization in OPCs. As shown in Figure 9, the immunostaining of OPCs treated with 2ME showed a reduction in α-tubulin staining compared to the untreated control. These inhibitory actions were also confirmed in the Western blotting of lysates from the control and 2ME-treated OPCs (Figure 9), suggesting that 2ME interferes with tubulin polymerization in OPCs.

**Figure 10:**
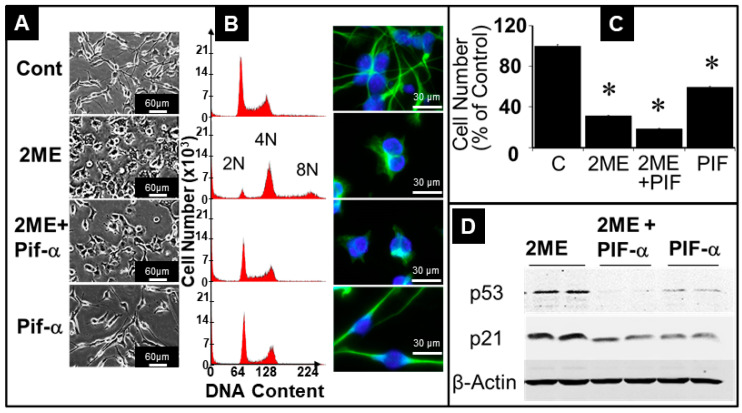
Inhibition of p53 with Pifithrin-α prevents 2ME-induced endoreduplication, but not arm process retraction, in Oli-neu cells. Bright-field microscopy ((**A**), magnification 4×) and FACS analysis (**B**) of Oli-neu cells treated with 5 µM 2ME for 18 h after pretreatment with or without Pifithrin-α 20 µM for 2 h. For FACS analysis, the DNA was stained with propidium iodide. Representative fluorescent images show 2ME-induced changes in Oli-neu morphology in the presence and absence of Pifithrin-α; magnification 100×. Bar graph depicts changes in Oli-neu cell number (**C**) and changes in the expression of p53 and p21 (**D**) in the presence and absence of Pifithrin-α. All experiments were repeated thrice and values in bar graph represent mean ± SEM (*n* = 3). * *p* < 0.05 vs. control.

We further analyzed the role of p53 signaling in 2ME-driven endoreduplication. We pretreated Oli-neu cells for 2 h with 20 µM Pifithrin-α, a reversible inhibitor of p53, and subsequently treated them with 5 µM 2ME in the presence of 20 µM Pifithrin-α for 24 h. Subsequently, the cells were fixed, stained with PI, and the DNA content was assessed by FACS analysis, as well as phase-contrast and bright-field microscopy. As shown in Figure 10A,B, treatment with 2ME induced the typical morphological changes in Oli-neu cells, which changed from an elongated and branched structure to a larger and more rounded shape. Treatment with 5 µM 2ME induced endoreduplication (Figure 10B; second row) with the generation of 8N cell population, whereas pretreatment with Pifithrin-α prevented the 2ME-induced endoreduplication (Figure 10B, third row), although the typical morphological changes induced by 2ME were not reversed (Figure 10, third row). The abrogatory effects of Pifithrin-α on 2ME-induced endoreduplication was also evident in cells evaluated microscopically. Treatment with 2ME, as well as Pifithrin-α, inhibited cell proliferation (Figure 10C) and the inhibitory actions were greater in OPCs treated with both 2ME and Pifithrin-α. Interestingly the inhibitory actions of Pifithrin-α on endoreduplication were accompanied by the downregulation of 2ME-induced p21 and p53 expression (Figure 10D). 

**Figure 11:**
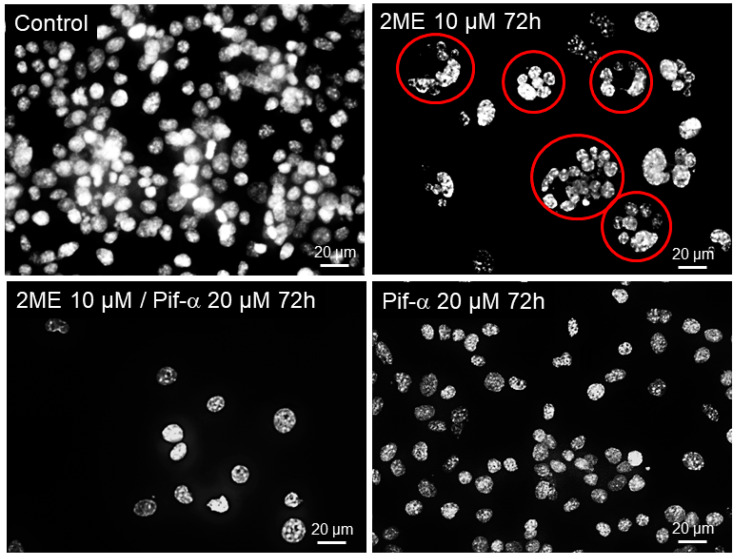
Representative fluorescent photomicrographs showing the Oli-neu clustering pattern following treatment with 5 µM 2ME for 72 h after pretreatment with or without Pifithrin-α 20 µM for 2 h. Inhibition of p53 prevented 2ME-induced clusters of multinucleated cells (red circles); magnification 40×.

The microscopic assessment of the OPCs also showed cells with multinucleated clusters (marked with red circles) prevalent in the 2ME-treated group, whereas this clustering was lost in cultures treated with 2ME in the presence of Pifithrin-α (Figure 11). 

### 3.9. 2ME Trigger Cell Fusion in OPCs

**Figure 12:**
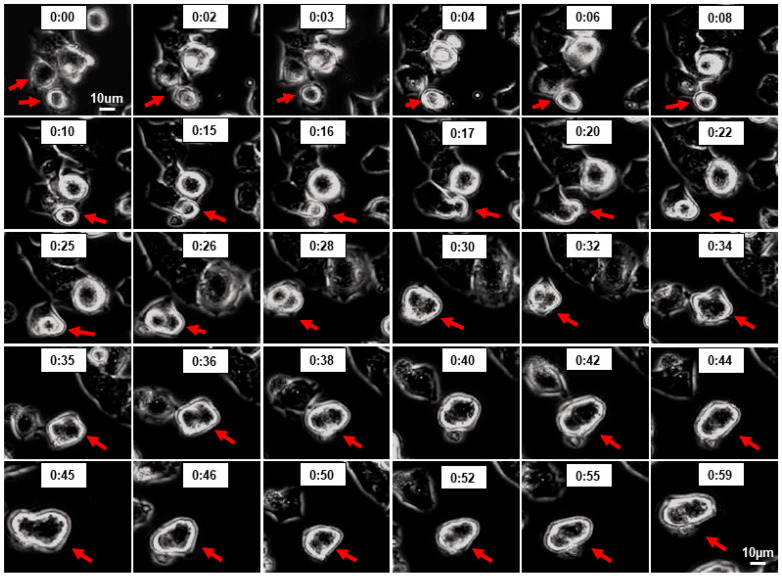
Time-lapse phase-contrast imaging showing 2ME-induced cell fusion in Oli-neu. Photomicrographs depict time sequence of two Oli-neu cells undergoing cell fusion (indicated by red arrows) following 2-day treatment of cultures with 5 µM 2ME. Similar effects were observed in four different experiments. (Time: seconds, magnification 100×.)

In addition to intracellular mechanisms, the fusion of cells may also contribute to multinucleated cells. It is well known that the cell-fusion process in cancer cells contributes to drug resistance, as has been observed for microtubule-interfering drugs. Since 2ME interacts with tubulin, it may also trigger cell fusion. As shown in Figure 12, in the live microscopy experiments, the treatment of Oli-neu cells with 5 and 10 µM triggered cell activity, with some cells actively migrating, interacting with neighboring cells, and undergoing cell fusion. Interestingly, the treatment with 2ME upregulated survivin in the Oli-neu cells (Figure 8), suggesting that 2ME triggers cell survival activities. Cell fusion activity was restricted to cells that were hyperactive or under duress and able to move/migrate, suggesting that under crisis for existence, OPCs can fuse to survive and resist.

### 3.10. Growth-Inhibitory Actions of 2ME in CG4 Cells

**Figure 13:**
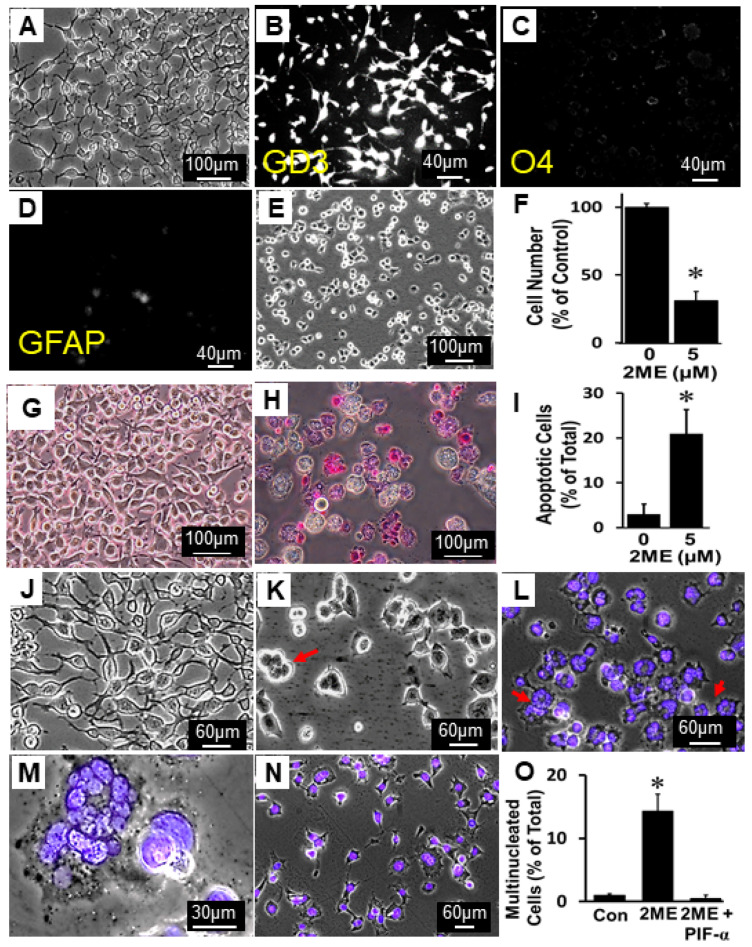
Representative photomicrographs and bar graphs depicting the growth-inhibitory and endoreduplication actions of 2ME in O-2A progenitor cells. Panel (**A**) shows phase-contrast photomicrograph of CG4 cells (magnification 100×) with bipolar morphology. The cells used for growth studies stained positive for GD4 (**B**) but were negative for O4 (**C**) and GFAP (**D**). Treatment with 5 µM 2ME for 24 and 48 h contracted bipolar arm extension (**E**) and inhibited cell proliferation (cell number (**F**)), respectively. Compared to untreated controls (**G**), treatment with 2ME for 20 h induced apoptosis, as assessed by APOPercentage dye ((**H**); magnification 40×). Bar graph in panel (**I**) depicts the percentage of apoptotic cells. Compared to untreated controls (**J**), treatment with 5 µM 2ME for 48 h increased the number of multinucleated cells (red arrows, panel (**K**), phase contrast 10×; panel (**L**), DAPI-stained 10×; panel (**M**), magnified image of multinucleated cells marked with red arrow in panel (**L**)). Pretreatment with 20 µM of the p53 inhibitor Pifithrin-α prevented 2ME-induced endoreduplication (panel (**N**), DAPI-stained photomicrograph 10×; panel (**O**), bar graph showing the number of multinucleated cells). All experiments were repeated thrice and values in bar graphs represent mean ± SEM (*n* = 3). * *p* < 0.05 vs. control.

To assess whether 2ME induces similar actions in other OPCs, we tested its impact on CG4 cells, which is a well-established progenitor cell line (oligodendrocyte-type-2 astrocyte, O-2A) shown to induce high-grade glioma [8,29]. The CG4 cells were cultivated in proliferation medium, where they are known to maintain the progenitor phenotype and do not differentiate. As shown in Figure 13, a homogenous population of bipolar cells was present (Figure 13A). Consistent with the characteristics of the OPCs, the cells used for the growth experiments stained positively for GD3 and were negative for O4 and GFAP (Figure 13B–D). Treatment with 2ME for 24 and 48 h led to shortening of the arms and the rounding of CG4 cells (Figure 13E), and inhibited cell proliferation (Figure 13F), respectively. Apoptosis studies, utilizing APOPercentage, demonstrated that 2ME (5 µM) induces apoptosis in CG4 cells, as depicted in Figure 13G (untreated control) and Figure 13H (2ME). The bar graph in panel I shows the increase in apoptotic cells in 2ME-treated CG4 cells. Compared to the untreated controls (Panel J), the treatment of CG4 cells with 5 µM 2ME for 48 h increased the number of multinucleated cells (Panels K–M), and these effects were abrogated in cells pretreated for 2 h with 20 µM Pifithrin-α, a p53 inhibitor (Figure 13N,O). These results are similar to our observations in Oli-neu OPCs, and suggest that 2ME can inhibit growth and induce endoreduplication in OPCs, potentially via p53.

## 4. Discussion

2-Methoxyestradiol and endogenous estradiol metabolites are effective against multiple proliferative disorders, including various cancer cells. In the present study, we demonstrate that 2ME treatment induced OPCs to be metabolically more active than the control cells and caused an accumulation of cells with a DNA content >4N, indicating that cells underwent endoreduplication. Our in vitro findings show that 2ME inhibits OPC growth, in part by inducing apoptosis and the upregulation of pro-apoptotic markers (caspase 3, caspase 7, and PPAR fragmentation; and phosphatidylserine externalization). Importantly, 2ME altered OPCs morphology by reducing their characteristic arm processes; moreover, at concentrations of >1 µM, 2ME induced endoreduplication in OPCs. The growth-inhibitory actions of 2ME were accompanied by the upregulation of p21 and p27 (negative regulators of cell cycle), whereas endoreduplication was accompanied by the significant upregulation of many cell-cycle proteins (cyclin A, cyclin B, cyclin D2, and cyclin E), as well as p53, a tumor suppressor known to cooperate with cyclin E and drive endoreduplication. At the signal transduction level, 2ME failed to influence MAPK phosphorylation; inhibited Akt phosphorylation; and induced pRb as well as JNK1/JNK2 phosphorylation. Interestingly, 2ME upregulated the expression of survivin and triggered OPC fusion. Importantly, we demonstrate that the inhibition of p53 with Pifithrin-α prevented 2ME-induced endoreduplication, but not morphological alterations (retraction of cell processes), suggesting that 2ME mediates endoreduplication via p53, whereas its morphological actions involve other mechanisms. Our findings provide the first evidence that 2ME not only inhibits OPC growth, but also triggers apoptotic and cell survival processes, leading to endoreduplication. We hypothesize that combining p53 inhibitors with MTIs may help prevent MTI-induced endoreduplication in cancer cells and be of clinical relevance.

Endoreduplication has been observed in the pathological evaluation of various cancers, including myomas. Moreover, it is prevalent in cells treated with MTIs, such as colchicine and nocodazole [30,31]. Our finding that 2ME, a tubulin-interfering agent, induced endoreduplication in OPCs is consistent with these observations. This supports the concept that MTIs can switch on a new replication cycle without anaphase chromosome segregation. Additionally, our data show that several cell-cycle proteins are regulated by 2ME treatment, and that the inhibition of p53 abrogated endoreduplication, indicating that microtubule inhibition leads to endoreduplication by a p53-dependent mechanism. 

Although polyploidy in mammals is evident in different mammalian cells under normal conditions (e.g., in trophoblast or myocardial cells), studies indicate that aneuploidy, genomic instability, and defects in cell-cycle checkpoints can contribute to tumorigenesis [30]. In this regard, it has been shown that aneuploidy is a characteristic of a great majority of human tumors [32]. Furthermore, it is linked to the progressive development of high-grade, invasive tumors [33,34], and it is a necessary intermediate in the formation of many solid human tumors [35]. Additionally, a high degree of aneuploidy correlates with poor patient prognosis [36,37]. Although polyploidy is observed in tumors and is linked to its progression, a cause-and-effect relationship remains unclear. Many MTIs known to induce polyploidy also inhibit tumor progression and cancer cell growth; hence, it is crucial to understand the mechanisms contributing to endoreduplication, and whether this leads to aneuploidy and tumorigenesis.

In the present study, we show that, at high concentrations, 2ME induces deleterious effects in OPCs. Considerable effort has been made to investigate whether 2ME could be used therapeutically to treat brain cancers, called gliomas, that originate from glial cells such as astrocytes and oligodendrocytes [38,39] by targeting their growth. Since gliomas are extremely difficult to treat and often fatal, new therapeutic molecules targeting their growth are required. Chemotherapy often fails in patients with gliomas due to their resistance to commonly used DNA-alkylating agents, and due to the activation of pro-survival pathways. The reasons why 2ME is a promising agent for the treatment of gliomas include the following: (1) 2ME is an MTI [40], and chemotherapy regimes that use MTIs such as vincristine are already used to treat gliomas [41]. (2) 2ME has pleiotropic antitumor activities, including both the inhibition of angiogenesis and cytotoxic effects on tumor cells. (3) Its mechanism does not rely on DNA alkylation. (4) It has been shown to be effective in an orthotopic rat glioma model. (5) It has moderate side effects in clinical phase I/II studies.

The above findings, together with our observation that 2ME induces polyploidy in OPCs and the fact that endoreduplication is linked to tumorigenesis, suggests that a better understanding of the cause-and-effect relationship between tumor/cancer growth and polyploidy/endoreduplication is required. Moreover, it is important to explore ways to reduce the risks of polyploidy development in response to drugs that interfere with microtubule dynamics. At a molecular level, endoreduplication results from an erroneous exit from mitosis due to a failure of the checkpoints, which normally regulate cell-cycle arrest at specific stages when previous events have not been completed. Although most cells sense the disturbance of the microtubule assembly after MTI treatment, some cells arrest only transiently, and thereafter exit mitosis without chromosome segregation and become tetraploid.

Consistent with our observations, 2ME has been shown to induce endoreduplication in both non-cancerous [42] and cancer cells [19,43,44]. The mechanism underlying 2ME-induced endoreduplication remains unclear, although its interaction with tubulin and the inhibition of polymerization seems to be involved. In the present study, we observed the inhibition of tubulin by 2ME in OPCs. Indeed, 2ME is known to be an MTI molecule [40] and endoreduplication has been observed in response to MTIs in cells lacking p53, pRb, and CdkIs p21 or p16 [45,46,47,48,49]. Moreover, in vascular smooth muscle cells, 2ME induced endoreduplication via a Cdk2-dependent pathway [43]. Interestingly, we also observed that 2ME inhibits growth and induces apoptosis, as well as endoreduplication, in another O-2A progenitor cell line, CG4.

Our results showed that p53 expression was regulated by 2ME. However, most studies so far indicate that p53 plays a major role in preventing endoreduplication by being a component of a spindle checkpoint that ensures the maintenance of diploidy. Data from several studies support the role of p53 in this mechanism: (1) p53 was shown to participate in a mitotic checkpoint that inhibits the formation of polyploid cells, since fibroblasts in cultures of p53-deficient mouse embryos exposed to spindle inhibitors formed tetraploid and octaploid cells [49]; (2) p53- and pRb-deficient fibroblasts re-replicate after treatment with the nocodazole, a strong MTI, whereas normal cells arrest in G1 [45,50]; (3) The abrogation of p53 in cooperation with Bcl-xL allowed rapid and progressive polyploidization following mitotic spindle damage [47].

In contrast to the above findings on p53, we observed that 2ME-induced endoreduplication was accompanied by p53 upregulation, and this was blocked by the p53 inhibitor Pifithrin-α. The reasons for the abrogation of endoreduplication by the p53 inhibitor in OPCs remains unclear. We hypothesize that p21 action may be responsible for this effect, as the p53-dependent inhibition of endoreduplication has been shown to induce p21 [46,48]. Moreover, the upregulation of p21 is known to negatively regulate cell-cycle progression at G1 and inhibit Oli-neu progression [51]. In oligodendrocytes, however, p21 has a controversial role, as p21 levels increase during S-phase entry [52].We speculate that the increase in p53 in response to 2ME leads to the expression of p21. Hence, instead of inhibiting the cell cycle, as in other mammalian cell types, this induces cells into S-phase entry, resulting in endoreduplication. This contention is supported by the fact that the inhibition of p53 by Pifithrin-α inhibited cell-cycle progression; moreover, treatment with Pifithrin-α alone decreased the percentage of cells in the S-phase compared to the control.

Consistent with our findings that p53 may be driving 2ME-mediated endoreduplication in OPCs, it has recently been reported that cyclin E-driven replicative stress promotes p-53-dependent mitotic bypass and whole-genome duplication (WGD) in p53-proficient cells [53]. This implies that tumor suppressor p53 can directly contribute to cancer evolution. Indeed, many genomics studies have shown that approximately half of the WGD events in cancer happen with a wild-type p53 background. Moreover, p53 has been shown to promote mitotic bypass after genotoxic or oncogene stress. However, how WGD happens in p53-proficient cells is still unclear. Zeng and colleagues [53] proposed that elevated cyclin E1, in concert with p53 and its downstream target p21, inhibits mitotic Cdk activity to promote mitotic bypass and complete endoreduplication. Consistent with this proposal, we observed a significant increase in both p53 and p21 and a minor change in cdk2.

Another potential mechanism leading to WGD is cell fusion. Interestingly, we observed increased metabolic and cell fusion activity in OPCs that were treated with 2ME. It is feasible that the pro-apoptotic actions of 2ME create a survival crisis in OPCs, thereby triggering vulnerable cells to find partners to resist and escape cell death. This may also contribute to the MTI drug resistance observed in cancer cells. Interestingly, in OPCs, 2ME induced the expression of survivin, which is a member of the chromosomal passenger complex implicated in cytokinesis, and modulates microtubule dynamics as well as nucleation [54]. Survivin plays an important role in the surveillance mechanism called mitotic spindle assembly checkpoint (MSAC), which regulates metaphase to anaphase transition during mitosis, potentially by interacting with p53 [55,56]. Interestingly, p53 is also linked to mitochondrial metabolic activity in cancer cells and regulates the balance between cell survival and death [57]. Finally, it is also possible that there are different populations of OPCs that express and react differently, as has been documented for microglial cells [58]. 

Our findings could have clinical relevance in deciding whether a glioma should be treated with an MTI or not. Moreover, it might be crucial to determine whether a glioma originates from oligodendrocytes or from another cell type in the brain, such as astrocytes. If the glioma is of oligodendroglial origin, chemotherapy with high concentrations of MTIs could lead to genomic instability and polyploidy. Our results suggest that polyploidy might be inhibited by Pifithrin-α; however, it is obvious that the inhibition of a major tumor suppressor protein in vivo might not be the solution to treat cancer cells.

Apart from tumor-suppressor proteins, cyclins critically regulate cell-cycle progression. One of the cyclins, cyclin B, is a direct target of the anaphase-promoting complex (APC). The APC plays an important role in the transition of cells from the metaphase to the anaphase [59]. The APC acts as a ubiquitin ligase and is required to label cyclin B for subsequent proteolysis, which in turn initiates the transition to the anaphase. It has been shown that 2ME enhances the amount of cyclin B in a tubulin-dependent manner, since cyclin B enhancement was absent in a tubulin-mutant clone [14 (55)]. The authors suggest that 2ME could induce mitotic arrest by inhibiting APC action. In our experiments, cyclin B was upregulated in response to 2ME. Although this should lead to a cell-cycle arrest, in 2ME-treated cells we did not observe a G1 arrest. However, the possibility that 2ME-treated cells were arrested in a pseudo G1 phase with an 8N DNA content cannot be excluded, as cell arrest in this phase could be accompanied by an increase in cyclin B expression.

Based on the above findings, 2ME treatment could result in cell-cycle arrest even in the presence of the induction of both cyclin B and p53 expression. Importantly, the upregulation of p53 and p21 may occur in non-arrested endoreduplicating cells, as observed here. Our data also showed that 2ME-treated cells had less pRB than control cells, but similar amounts of ppRb. Therefore, the percentage of pRb compared to total Rb was less in 2ME-treated cells than in control cells. It is known that pRb binds to E2F, thereby hindering the initiation of gene transcription. Since endoreduplication was associated with less pRb in Oli-neu cells, we suggest that the absence of pRb induced E2F to initiate S-phase transition, despite tetraploidy. Our findings indicate that pRb is an important factor in the cellular response to MTIs, particularly in the context of the regulation of DNA replication. Indeed, several studies have shown that pRb plays an important role in cell-cycle arrest after tetraploidy and that a functional pRb is crucial for ensuring appropriate cell-cycle arrest [45,46,50,60]. Interestingly, we see that 2ME upregulates JNK1, an important up-stream activator of cyclin B1 and cdc2, and is responsible for 2ME-induced endoreduplication in human breast cancer cells [19].

We also observed that 2ME treatment regulated the expression of cyclin E, which is a regulatory subunit of Cdk2 and drives cells from G1 to the S phase. Increased cyclin E expression is frequently observed in human malignancies and is associated with poor prognosis in various cancers. Under quiescent conditions, cyclin E forms a complex with Cdk2, which inhibits the phosphorylation of pRb and prevents the release of E2F, thereby inhibiting the entry of cells into the S phase. 2ME has been shown to induce endoreduplication in human smooth muscle cells, and the role of Cdk2 has been proposed as the cause [43]. In our experiments, however, we observed a minor increase in Cdk2 expression. In another study on MTI-induced endoreduplication, it was shown that the p21 inhibition of cyclin E/Cdk2 activity prevented endoreduplication after mitotic spindle disruption and, similar to our findings, the Cdk2 levels remained constant [48]. In that study, the expression of cyclin E was also investigated and, after an initial decline, probably due to loss of cells in the G1 state, cyclin E levels increased, consistent with the presence of cells in a pseudo G1 state. We speculate that cells undergo endoreduplication after contact with MTIs, either by upregulating Cdk2 or by increasing cyclin E expression. Additionally, the expression of cyclin E could be linked to the expression of p21, since the increased expression of p53 transactivates p21, which binds to the cyclin E/Cdk2 complex and thereby inhibits the progression to the S phase.

In addition to cyclin B and E, 2ME treatment also increased the expression of cyclin A, a suggested target for oncogenic signals that is associated with the E2F transcription factor. A study by Pagano et al. [61] demonstrated that cyclin A has two distinct kinase activities, one appearing in the S phase, the other in G2. Cyclin A was found to bind to both Cdk2 and Cdc2 cyclin A complexes with p21 and PCNA. However, no connection between cyclin A and endoreduplication has been found in mammalian cells so far [62], and could potentially be ruled out. 

2ME treatment also upregulated cyclin D2 expression. In nocodazole (another strong MTI)-treated HCT116 cells [48], cyclin D expression was analyzed in a time-dependent manner. A small decrease in cyclin D1 protein levels was observed after 18 h of treatment, consistent with the loss of G1 cells and the accumulation of cells in G2/M. Nevertheless, the cyclin D1 protein levels increased again after 32 h of treatment, which was consistent with the presence of cells in a G1-like biochemical state. In our FACS experiments, we found an accumulation of cells with 8N after 48 h of treatment with 2ME. The 8N peak in the FACS diagram tails on the right towards zero, indicating that cells might be arrested in a pseudo-G2 state. Interestingly, in our experiments, we observed that cells pretreated with Pifithrin-α and treated with 2ME showed the typical morphological alterations of MTI treatment, such as the retraction of cell processes and rounding. Interestingly, these cells did not undergo endoreduplication. Since 2ME treatment is known to induce spindle aberrations [39,63], we speculate that the round morphology of 2ME-treated cells was due to a destruction of the microtubule network, which causes the failure of the spindle apparatus. We speculate that there is a mechanism that senses the failure of spindle function in 2ME- and Pifithrin-α-treated cells and forces the cells to arrest. However, when p53 is not inhibited, the mechanism is abrogated or overridden by the presence of p53. Finally, other mechanisms such as HIF-1α may also be involved, as the inhibitory effects of 2ME on glioma growth were accompanied by the inhibition of HIF-1α [22]; however, this could not be tested in our study.

## 5. Conclusions

Our findings provide evidence that at concentrations greater than 1 µM, 2ME not only induces apoptosis, but also activates OPCs and triggers endoreduplication, potentially via p53. Hence, the therapeutic use of 2ME as an antitumor/anticancer agent may face the same fate as other microtubule-interfering drugs. Since Pifithrin-α, a p53 inhibitor, prevented 2ME-induced endoreduplication, combining MTI treatments with p53 inhibitors may prevent endoreduplication and potentially MTI drug resistance in gliomas. Our results provide important leads; however, future in-depth studies are required to confirm these possibilities.

**Limitations**: The focus of our study was to assess the inhibitory potential of 2ME in OPCs, which contribute to glioma pathology. Our finding that 2ME inhibits OPC growth and triggers endoreduplication and OPC arm retraction at pharmacological concentrations in vitro would need to be confirmed in an in vivo setting, as 2ME pharmacokinetics may differ between in vitro and in vivo conditions. More importantly, experiments must be conducted under an appropriate O2 concentration for OPCs isolated from tumors, and the actions of 2ME on tumor-derived and normal OPCs should be compared to gain better insights regarding its therapeutic potential.

## Data Availability

All data supporting the findings of this study are available within the article and its Supplementary Files or from the corresponding author upon reasonable request.

## Supplementary Materials

The following supporting information can be downloaded at: https://www.mdpi.com/article/10.3390/cells13131086/s1, Table S1: Materials used.
