# Supplementary material for: 2-Methoxyestradiol, an Endogenous 17β-Estradiol Metabolite, Induces Antimitogenic and Apoptotic Actions in Oligodendroglial Precursor Cells and Triggers Endoreduplication via the p53 Pathway
Source: Cells. 2024 Jun 22;13(13):1086. doi: 10.3390/cells13131086 (PMC11240791; doi:10.3390/cells13131086)
Supplement: Supplementary file 1 [file cells-13-01086-s001.zip › cells-2993889-supplementary Table S1.pdf]

# Material:

## Cell culture

| | |
|---|---|
| Antibioticum-Antimycotium (AA) | GIBCO BRL, Paisley, UK (15240-096) |
| Dulbecco's Modified Eagle Medium (DMEM) | GIBCO BRL, Paisley, UK (13960-044) |
| Dulbecco's Modified Eagle Medium (DMEM)-F12 | GIBCO BRL, Paisley, UK (21331-020) |
| FCS Charcoal Stripped | Hyclone, MA, USA (SH30068.03) |
| Fetal Calf Serum (FCS) | GIBCO BRL, Paisley, UK (16140-071) |
| Gentamicine | Sigma-Aldrich, Buchs,CH (G-1272) |
| Hank`s Buffered Salt Solution with $Ca^{2+}$ and $Mg^{2+}$ | Sigma-Aldrich, Buchs,CH (H-8294) |
| Hank`s Buffered Salt Solution without $Ca^{2+}$ and $Mg^{2+}$ | Bioconcept, Allschwil, CH (3-02K34-I) |
| Insulin | Sigma-Aldrich, Buchs,CH (I-9278) |
| L-Glutamine | Sigma-Aldrich, Buchs,CH (G-7513) |
| L-Thyroxine | Sigma-Aldrich, Buchs,CH (T-1775) |
| Oli-neu Cells (Oligodendrocytes) | From Martin Moransard, BZE UZH/ETH |
| Poly-D-Lysine | Sigma-Aldrich, Buchs,CH (P-7820) |
| Progesterone | Sigma-Aldrich, Buchs,CH (P-0130) |
| Putrescine | Sigma-Aldrich, Buchs,CH (P-7505) |
| Sodium Selenite | Sigma-Aldrich, Buchs,CH (S-5261) |
| Transferrin | Sigma-Aldrich, Buchs,CH (T-2252) |
| Tri-Iodo-Thyrodine | Sigma-Aldrich, Buchs,CH (T-6397) |
| Trypsin | Sigma-Aldrich, Buchs,CH (T-3924) |

## Antibodies and Peptides

| | |
|---|---|
| Anti.Survivin | Chemicon International, CA, USA (AB3610) |
| Anti-Akt | Cell Signaling Technology, MA, USA (9272) |
| Anti-Caspase 3 | Upstate Biotechnology, NY, USA (06-735) |
| Anti-Caspase7 | Cell Signaling Technology, MA, USA (9492) |
| Anti-cdk2 | Upstate Biotechnology, NY, USA (06-505) |
| Anti-Cleaved Caspase 3 | Cell Signaling Technology, MA, USA (9661) |
| Anti-Cleaved Caspase 7 | Cell Signaling Technology, MA, USA (9491) |
| Anti-Cyclin B1 | Cell Signaling Technology, MA, USA (4138) |
| Anti-Cyclin D | Millipore, Temecula, CA (06-137) |
| Anti-Cyclin E | Cell Signaling Technology, MA, USA (4129) |
| Anti-p21 | BD Biosciences, Europe (554228 |
| Anti-p27 | Chemicon International, CA, USA (AB3003) |
| Anti-p38 MAPK | Cell Signaling Technology, MA, USA (9212) |
| Anti-PARP | Cell Signaling Technology, MA, USA (9542) |

| | |
|---|---|
| Anti-Phospho-Akt (Ser473 | Cell Signaling Technology, MA, USA (9271) |
| Anti-Phospho-p38 MAPK | Cell Signaling Technology, MA, USA (9211) |
| Anti-Phospho-SAPK/JNK | Cell Signaling Technology, MA, USA (9255) |
| Anti-pRb | BD Biosciences, Europe (554136) |
| Anti-SAPK/JNK | Cell Signaling Technology, MA, USA (9252) |
| Anti-β-Actin | Sigma, St Louis, USA (A-5441) |
| IGF-Peroxidase Conj. Goat anti-Mouse | Pierce, Rockford, USA (31430) |
| IGF-Peroxidase Conj. Goat anti-Rabbit | Pierce, Rockford, USA (31460) |
| IRDye 680 Conj. Goat anti-Mouse IgG | LI-COR, NE, USA (926-32220) |
| IRDye 680 Conj. Goat anti-Rabbit IgG | LI-COR, NE, USA (926-32221) |
| IRDye 800 Conj. Goat anti-Mouse IgG | LI-COR, NE, USA (926-32210) |
| IRDye 800 Conj. Goat anti-Rabbit IgG | LI-COR, NE, USA (926-32211) |

**Chemicals and Buffers**

| | |
|---|---|
| 2-Methoxyestradiol (2ME) | Steraloids, Newport. RI, USA (E2490) |
| 5'-$^{3}$H-Thymidine | Perkin Elmer NEN, Wellesley, USA (NET027Z001MC) |
| DAPI counterstain | Vysis Inc. (32-804 831) |
| Propidium iodide (PI) | Sigma, St. Louis, USA (P-4170) |
| Thiazol Blue Tetrazolium Bromide (MTT) | Sigma-Aldrich, MO, USA (M-5655) |
